# A meta-analysis: the clinical value of PD-1 inhibitor or protein tyrosine kinase inhibitors in the treatment of advanced osteosarcoma

**DOI:** 10.3389/fonc.2023.1148735

**Published:** 2023-06-12

**Authors:** Binhao Shi, Junli Chang, Xingyuan Sun, Xiaoping Ma, Peng Zhao, Chujie Zhou, Yongjun Wang, Yanping Yang

**Affiliations:** ^1^ Longhua Hospital, Shanghai University of Traditional Chinese Medicine, Shanghai, China; ^2^ Key Laboratory of Theory and Therapy of Muscles and Bones, Ministry of Education, Shanghai, China

**Keywords:** immunotherapy, PD-1 inhibitors, protein tyrosinase inhibitors, advanced osteosarcoma, meta-analysis

## Abstract

**Backgrounds:**

PD-1 inhibitors and TKIs have been used to treat advanced osteosarcoma, but there is still a lack of intuitive data for the comparison of their efficacy. We conducted a meta-analysis to evaluate their therapeutic benefits.

**Methods:**

A systematic methodological search of five primary electronic databases was performed. Studies with a randomized design of any type about PD-1 inhibitors or TKIs for the treatment of advanced osteosarcoma were included. The primary outcomes mainly included CBR, PFS, OS and ORR, The CR, PR, SD and AEs were the secondary outcomes. The survival period (months) of patients was taken as the main analysis data. Random-effects models were used for meta-analysis.

**Results:**

Eight immunocheckpoint inhibitors in 327 patients from 10 clinical trials were finally evaluated. For OS, TKIs [11.67 months (95% CI, 9.32-14.01)] show more obvious advantages than PD-1 inhibitors [6.37 months (95% CI, 3.96-8.78)]. For PFS, TKIs [4.79 months (95% CI, 3.33-6.24)] are longer than PD-1 inhibitors [1.46 months (95% CI, 1.23-1.69)]. Although there was no fatal event, attention should still be paid, especially during the combined application of PD-1 inhibitors with TKIs since their obvious AEs.

**Conclusions:**

The findings of this study suggest that patients with advanced osteosarcoma, TKIs may be more beneficial than PD-1 inhibitors. TKIs combined with PD-1 inhibitors has a bright future in the treatment of advanced osteosarcoma, but we should always pay attention to the strong side effects.

## Introduction

Osteosarcoma is a malignant bone tumor that often occurs in the metaphysis of growing long tubular bones and usually arises in children, adolescents and young adults (1, 2), which is easy to invade the lungs and other bones. Modern imaging techniques identify that about 25% of newly diagnosed patients showing metastatic lesions (3). Local surgery and chemotherapy are still currently the dominant treatment strategy for the osteosarcoma patients, with the combination of high dose methotrexate, adriamycin and cisplatin (MAP) as the international standard regimen in perioperative period (4). This greatly contribute to the 5-year overall survival (OS) rate and the 5-year event-free survival rate of patients after successful surgery and chemotherapy reaching about 60% (1). However, patients with initially metastatic disease, those who relapse after achieving remission, or those who fail to respond to the MAP regimens have an extremely poor 5-year survival rate of less than 30% (5). Furthermore, approximately one third of osteosarcoma patients will eventually develop disease recurrence or metastasis (6). Moreover, the efficacy of chemotherapy combination in the treatment of advanced osteosarcoma is limited, with an effective rate of only 3% to 29% and a median PFS of less than 4 months (7–9).

Immunotherapy is a method to improve the immune clearance of cancer cells via complementation or stimulation of the immune system with a plethora of compounds, which includes the three main forms of cancer vaccines, cytokine therapies and passive cancer immunotherapy (10). As an important form of tumor immunotherapy, immune checkpoint inhibitors bring hopes to patients with refractory tumors and have improved the prognosis of many patients with advanced tumors (11, 12), which belongs to the passive cancer immunotherapy. Programmed cell death protein (PD-1) is a member of the immunoglobulin gene superfamily, and exerts the immunosuppressive effect on active T cells. Interaction between PD-1 with programmed death-ligand 1 (PD-L1) results in tumor-specific T cell exhaustion and apoptosis, which enables tumor cells to evade the T cell based immune surveillance. Theoretically, inhibition of PD-1 relieves the immunosuppressive microenvironment of activated T cells, B cells, monocytes, dendritic cells (DCs), regulatory T cells (Tregs) and natural killer T cells (NKTs), thus to improve the killing effect on tumor cells and inhibit the tumor growth (13). For example, nivolumab and pembrolizumab showed anti-tumor activity in the treatment of advanced osteosarcoma. However, the current clinical benefits have not met people’s expectations (14, 15).

As a major member of the protein kinase family, protein tyrosine kinases play vital roles in the growth, regulation, differentiation, migration, and apoptosis of tumor cells. Excessive tyrosine kinase activity induces the initiation and progression of malignancy (16). Therefore, ideally, the malignant tumor related tyrosine kinase can be used as an inhibitory target to control the continuous development of the malignant tumors. Multiple potent and well-tolerated tyrosine kinase inhibitor (TKI)-targets, including EGFR (epidermal growth factor receptor), ALK (anaplastic lymphocyte kinase), ROS1 (ROS proto-oncogene 1), HER2 (human epidermal growth factor receptor 2), TRK (tropomyosin receptor kinase), VEGFR (vascular endothelial growth factor receptor), RET (rearranged during transfection), MET (mesenchymal-epithelial transition factor), MEK (mitogen-activated protein kinase pathway), FGF (fibroblast growth factor), PDGF (Platelet-derived growth factor), and KIT (proto-oncogene c-KIT), have emerged and contributed to the significant improvement in cancer treatment (17). Tyrosine kinase inhibitors (TKIs) have objective responsiveness in the treatment of osteosarcoma, such as retinofenib and cabozantinib (18). In addition, the positive expression of VEGFR-2 and PD-L1 is significantly correlated with the grading and distant metastasis of osteosarcoma. VEGF/VEGFR inhibitors can play a synergistic role with PD-1 inhibitors in the treatment of osteosarcoma (15).

PD-1 inhibitors and TKIs have been used in patients with inoperable or recurrent osteosarcoma. However, there is no systematic evaluation of the difference between PD-1 inhibitors and TKIs in inhibiting disease progression and improving the survival of patients with advanced osteosarcoma. Our current work attempts to compare the efficacies of PD-1 inhibitors and TKIs in advanced osteosarcoma through a comprehensive meta-analysis.

## Search methods and study selection

To identify the therapeutic effects of PD-1 inhibitors or TKIs on osteosarcoma, a comprehensive literature search for the published clinical trials was performed from December 2, 2021, through final search for updates on May 30, 2022, in PubMed, Web of Science, Embase, Science Direct, Scopus and Cochrane library using the terms of osteosarcoma, bone sarcoma, osteogenic sarcoma, PD-1 inhibitor, PD-L1 inhibitor, anti-PD-1, anti-PD-L1, TKIs, and tyrosine kinase inhibitor.

The included studies met all the following criteria: (1) clinical trial of osteosarcoma treatment, (2) participants were treated with a single drug PD-1 inhibitor or TKI, (3) reported the treatment-related data, and (4) published in English. Exclusion criteria: (1) Literature review; (2) Non clinical trial literature; (3) Non English literature. Literature search, study selection and data extraction were conducted by two independent researchers, and discrepancies were reviewed by another researcher in the team and resolved by consensus. Using the MINORS scale or JADAD scale to evaluate the quality of included literatures. The review was registered with PROSPERO (CRD42022297820). This study followed the Preferred Reporting Items for Systematic Reviews and Meta-analyses (PRISMA) guideline.

### Data extraction

The first author, the year of publication, the country, the trial name, the phase, the name of medicine, the number of patients, the staging, the efficacy estimation indices (CBR, ORR, PFS, OS). Grade 3 or higher (severity) adverse event (AE) data were both extracted.

### Statistical analysis

The number of events and the total number of participants in the clinical trials were obtained. The relative risk and 95% CI were calculated using Stata 14 software. Heterogeneity of studies supporting each pairwise comparison was also evaluated quantitatively using chi-square test for heterogeneity and the inconsistency statistic (I^2^). If *P* > 0.1, I^2^<50%, it means that there is no statistical heterogeneity among the research results, and the fixed effect model is used for combined analysis. If *P ≤* 0.1, I^2^≥50%, it indicates that there is statistical heterogeneity among the research results, and the random effect model is used for combined analysis.

## Results

### Eligible studies and characteristics

**Figure 1:**
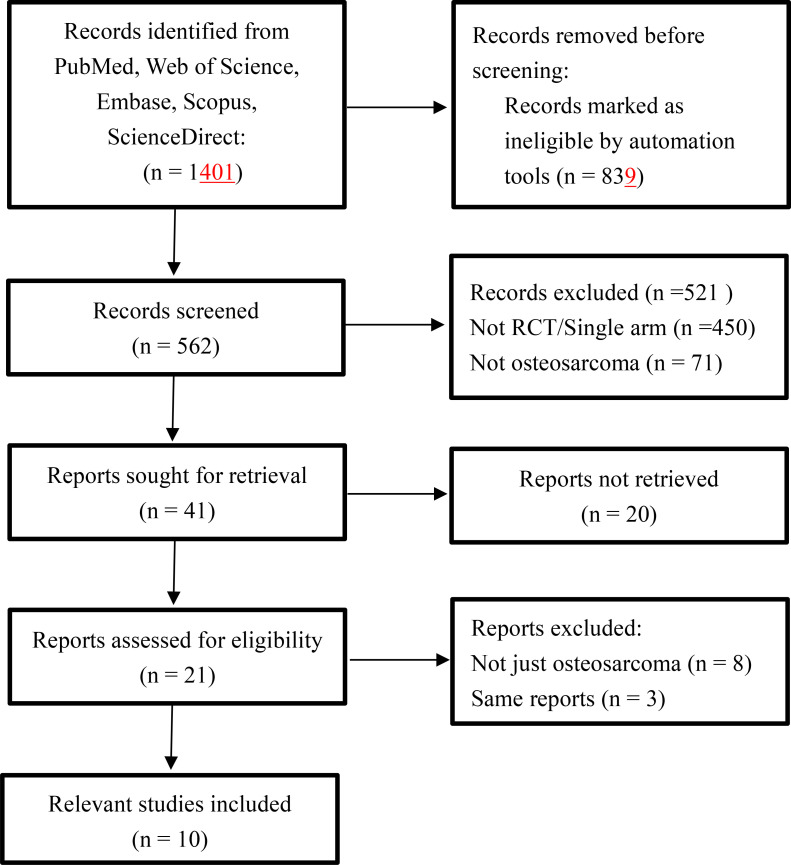
Flow diagram for the meta-analysis.

A total of 1401 publications were identified after literature search and browsing. Ten clinical trials involving 327 patients were finally included in this meta-analysis after the screening and qualification evaluation (**Figure 1**; **Table 1**). PD-1 and PD-L1 inhibitors for treatment included Pembrolizumab and Camrelizumab. TKIs for treatment included Regorafenib, Lenvatinib, Apatinib, Regorafenib, Cabozantinib, and TAS-115.

**Table 1:** Characteristics of the trials.

Author	Year of publication	Country	Study Design	Patient No.	Stage	Age, Mean (Range)	Drug attribution	Drug name	Median Follow-up (month)	Primary endpoint	Secondary endpoint
Boye K, et al. (19)	2021	Norway	single-arm, open−label, phase II	12	advanced	43 (19-55)	anti-PD-1	Pembrolizumab	—	CBR,PFS,OS	CR, PR, SD, AEs
Le Cesne A, et al. (20)	2019	France	multicenter, single-arm, phase II	17	advanced	41 (18-84)		Pembrolizumab+cyclophosphamide	18.9	PFS,OS	CR, PR, SD, AEs
Davis LE, et al. (21)	2019	USA	multicenter, randomized, double-blind, phase II	42	advanced	37 (18-76)	TKIs	Regorafenib	7.4	PFS,OS	AEs
Gaspar N, et al. (22)	2021	France	multicenter, open-label, single-agent	31	advanced	15 (9-22)		Lenvatinib	16.6	CBR,ORR,PFS,OS	PR, SD, AEs
Xie L, et al. (23)	2019	China	open-label, phase II	37	advanced	23 (16-62)		Apatinib	4.5	CBR,ORR, PFS,OS	CR, PR, SD, AEs
Duffaud F, et al. (24)	2019	France	non-comparative, double-blind, controlled, phase II	38	advanced	33 (22-50)		Regorafenib	31.6	PFS,OS	CR, PR, SD, AEs
Italiano A, et al. (25)	2020	France	multicenter, single-arm, phase II	45	advanced	34 (20-53)		Cabozantinib	31.1	PFS,OS	CR, PR, SD, AEs
Kawai A, et al. (26)	2021	Japan	multicenter, open-label, phase I	20	advanced	30 (16-64)		TAS-115	—	ORR, PFS	CR, PR, SD, AEs
Gaspar N, et al. (27)	2021	France	multicenter,open-labe, multicohort, phase I/II	42	advanced	15 (12-19)		Lenvatinib with etoposide + ifosfamide	9.6	CBR,ORR,PFS,OS	CR, PR, SD, AEs
Xie L, et al. (28)	2020	China	single-arm, open-label, phase II	43	advanced	19 (11-43)	TKIs+ anti-PD-1	Apatinib+Camrelizumab	11.3	CBR,ORR,PFS,OS	CR, AEs

PD-1, programmed cell death protein 1; TKIs, tyrosine kinase inhibitors; CBR, clinical benefit rate; PFS, progression-free survival; OS, overall survival; CR, complete response; PR, partial response; SD, stable disease; ORR, overall response rate; AEs, adverse events (drug-related, grade 3 or higher).

Among the 10 clinical trials included, two were randomized controlled double-blind trials, and the rest were single arm trials. There were two literatures on the treatment of osteosarcoma with PD-1 inhibitors, seven literatures on the treatment of osteosarcoma with TKIs, and one literature on the treatment of osteosarcoma with PD-1 inhibitors combined with TKIs. The quality of all 10 articles meets the inclusion requirements (**Table 2**). Among the 327 patients included, 132 were females and 195 were males, involving Europeans and Americans (Norway, France and the United States) and Asians (China, Japan). All the patients were in advanced stage of osteosarcoma. The efficacy evaluation indexes mainly included CBR (clinical benefit rate), PFS (progression free survival), OS (overall survival), CR (complete response), PR (partial response), SD (stable disease), ORR (overall response rate), AEs (adverse events). In view of the quality of literature data, we only performed statistical analysis on OS and PFS (**Table 3**). Next, we conducted the sensitivity analysis to evaluate the selected literatures. The results showed that OS related literatures (I^2^ = 61.7%, *P* = 0.007) and PFS related literatures (I^2^ = 93.7%, *P* = 0.000). Subgroup analysis by drug grouping was then conducted to objectively evaluate the difference between the two immune checkpoint inhibitors.

**Table 2:** Literature quality evaluation assessment.

Author	Study Design	MINORS	JADAD
Boye K, et al. (2021) (19)	single-arm, open−label, phase II	12	
Le Cesne A, et al. (2019) (20)	multicenter, single-arm, phase II	14	
Davis LE, et al. (2019) (21)	multicenter, randomized, double-blind,phase II		4
Gaspar N, et al. (2021) (22)	multicenter, open-label, single-agent	14	
Xie L, et al. (2019) (23)	open-label, phase II	12	
Duffaud F, et al. (2019) (24)	non-comparative, double-blind, controlled, phase II		7
Italiano A, et al. (2020) (25)	multicenter, single-arm, phase II	14	
Kawai A, et al. (2021) (26)	multicenter, open-label, phase I	12	
Gaspar N, et al. (2021) (27)	multicenter,open-labe, multicohort, phase I/II	14	
Xie L, et al. (2020) (28)	single-arm, open-label, phase II	14	

**Table 3:** Characteristics of the endpoints.

Author	Drug attribution	Drug name	Patient No.	Primary endpoint				Secondary endpoint			
				CBR	ORR	OS(M)	PFS(M)	CR	PR	SD	AEs(≥III)
Boye K, et al.	anti-PD-1	Pembrolizumab	12	0	0	6.6	1.7	0	0	0	0
Le Cesne A, et al.		Pembrolizumab + cyclophosphamide	17	2/15	1/15	5.6	1.4	0	1	5	7
Davis LE, et al.	TKIs	Regorafenib	42	—	—	11.1	3.6	—	3	—	6
Gaspar N, et al.		Lenvatinib	31	15/31	6.7%	10.0	3.0	—	2	13	9/31
Xie L, et al.		Apatinib	37	13/37	16/37	9.87	4.5	0	16	8	19
Duffaud F, et al.		Regorafenib	38	—	—	11.3	3.8	0	2	0	7/29
Italiano A, et al.		Cabozantinib	45	—	7/42	10.6	6.7	0	7	26	61/90
Kawai A, et al.		TAS-115	20	—	0	—	3.0	0	0	10	17/20
Gaspar N, et al.		Lenvatinib with etoposide + ifosfamide	42	13/35	3/32	16.3	8.7	0	3	10	31/42
Xie L, et al.	TKIs+ anti-PD-1	Apatinib + Camrelizumab	43	30.2%	20.1%	11.3	6.2	0	—	—	30/43

CBR, clinical benefit rate; ORR, overall response rate; OS, overall survival; PFS, progression-free survival; CR, complete response; PR, partial response; SD, stable disease; AEs, adverse events (drug-related, grade 3 or higher).

### The OS in osteosarcoma patient treatment with PD-1 inhibitors or TKIs

**Figure 2:**
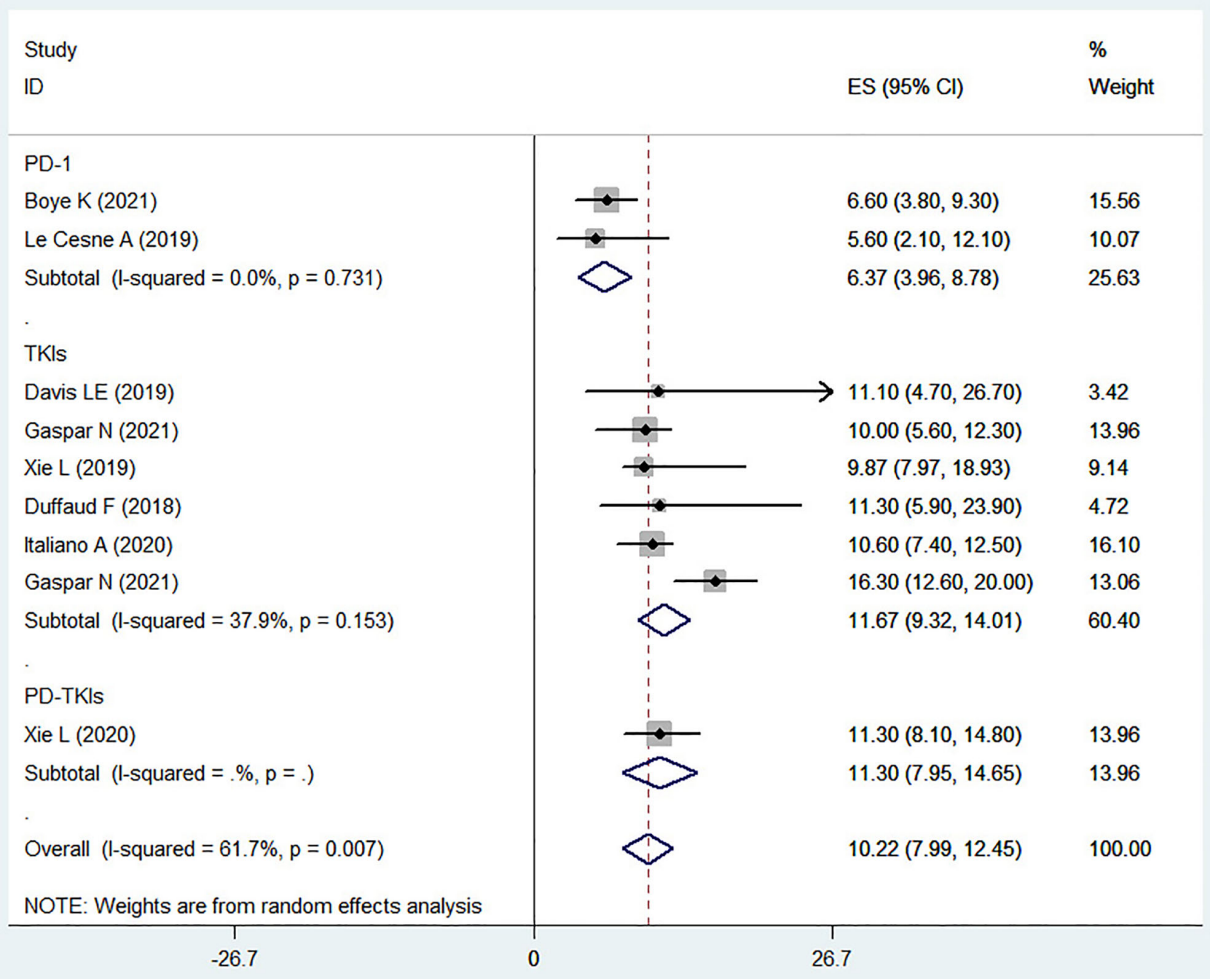
The OS of osteosarcoma patients treated with PD-1 inhibitors or TKIs.

The OS data were available from 9 (of 10) studies including 307 patients. The statistical analysis of all literatures suggested that the total OS was 10.22 months (95% CI, 7.99-12.45). Two clinical trials of PD-1 inhibitors (I^2^ = 0.0%, *P* = 0.731) showed that the combined OS of PD-1 inhibitors in the treatment of advanced osteosarcoma was 6.37 months (95% CI, 3.96-8.78) (**Figure 2**), which was similar to their respective median OS [6.60 months (95% CI, 3.80-9.30) or 5.60 months (95% CI, 2.10-12.10)]. The PD-1 inhibitors used in both studies were pembrolizumab. Six trials on TKIs (I^2^ = 37.9%, *P* = 0.153) showed that the OS of TKIs in the treatment of advanced osteosarcoma was 11.67 months (95% CI, 9.32-14.01). The TKIs used in these clinical trials included Lenvatinib, Cabozantinib, Regorafenib and Apatinib. The OS of each clinical trial was 11.1 months (95% CI, 4.7-26.70), 10.00 months (95% CI, 4.7-26.70), 9.87 months (95% CI, 7.97-18.93), 11.3 months (95% CI, 5.90-23.9), 10.60 months (95% CI, 7.40-12.50), or 16.30 months (95% CI, 12.60-20.00). Only one clinical trial applied the PD-1 inhibitor combined with TKI in the treatment of advanced osteosarcoma, which showed that the OS was 11.30 months (95% CI, 7.95-14.65). From the statistical results of drug classification, compared with PD-1 inhibitors, it seems that the survival period of advanced osteosarcoma patients treated with TKIs have been prolonged.

### The PFS in osteosarcoma patient treatment with PD-1 inhibitors or TKIs

PFS was systematically described in the ten included clinical trials. A total of 327 patients with advanced osteosarcoma were included for the final statistical analysis, which evidenced that the PFS in the advanced osteosarcoma patient treatment was 1.46 months (95% CI, 1.23-1.69) for the PD-1 inhibitors, 4.79 months (95% CI, 3.33-6.24) for the TKIs, and 6.20 months (95% CI, 4.75-7.65) for the PD-1 inhibitors combined with TKIs.

**Figure 3:**
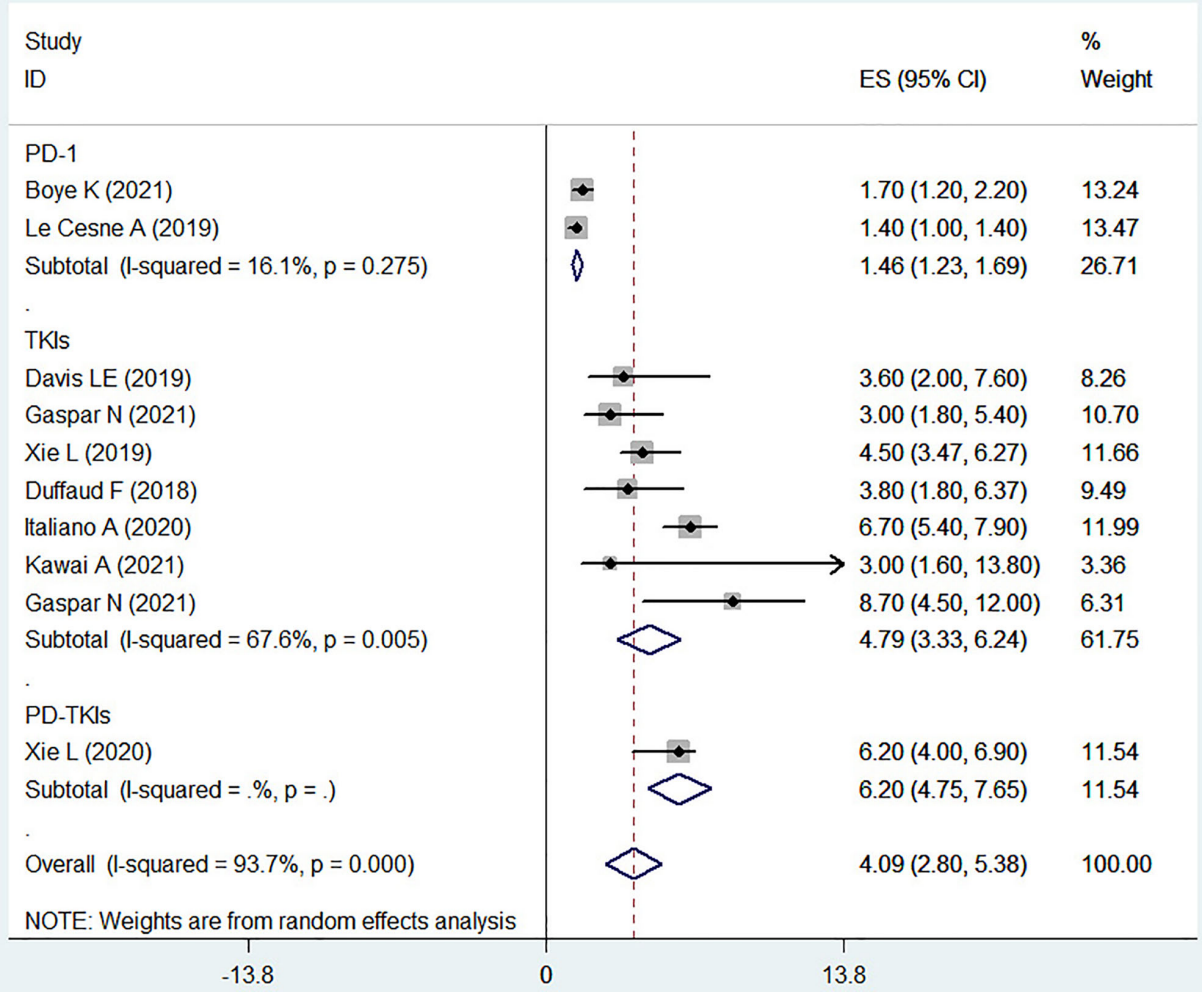
The PFS of osteosarcoma treated with PD-1 or TKIs.

The included clinical trials with TKIs as the test drug showed a significant heterogeneity (I^2^ = 67.6%, *P* = 0.005), indicating that different test drugs may have different impact on the PFS, and the overall median PFS improved by PD-1 inhibitors combined with TKIs was more meaningful (**Figure 3**).

### The AEs in osteosarcoma patient treatment with PD-1 inhibitors or TKIs

The immune related adverse events (irAEs) refer to the immune activation and inflammatory response against the healthy tissues of the hosts, which are the common concomitant phenomena of immunotherapies. The drug-related adverse reactions over grade III in the included literatures were sorted out to evaluate the AEs(**Table 4**). Pembrolizumab may cause drug-related AEs such as anemia, fatigue, leuopenia, acute renal failure, dyspnea, lymphoenia (13). Camrelizumab may lead to hypothyroidism, thrombocytopenia, anorexia and diarrhea (25). The severity of the treatment-related AEs in the Regorafenib was at least grade III, such as hypertension, hypophosphataemia, hand-foot skin reaction, increased transaminases, increased lipase, increased blood alkaline phosphatase, epilepsy, haemothorax and fatigue (14, 21). Lenvatinib may cause hypertension, pneumothorax, colonic perforation, anemia, thrombocytopenia, febrile neutropenia and neutropenia (19, 24). Grade III or above AEs during the treatment of osteosarcoma patients with Apatinib included hypertriglyceridemia, increased blood bilirubin, increased transaminases, increased blood LDH, weight loss, leukopenia, decreased platelet count, pneumothorax, wound dehiscence, diarrhea, anorexia, hand-foot syndrome, pain in extremity, rash, mucositis oral, hypertension, abdominal pain, toothache, non-cardiac chest pain, proteinuria, cough, nausea, vomiting, hemorrhoidal hemorrhage, fatigue, peripheral neuroinflammation, abdominal cramps, pleural infection, bladder perforation, anemia, hypokalemia, palpitations, back pain, anorectal infection and cholecystitis (20, 25). The increased transaminases, increased lipase, hypertension, thrombocytopenia, hypophosphatemia, neutropenia, hypomagnesaemia, leucopenia, fatigue, diarrhea, oral mucositis, palmar-plantar syndrome, anemia, pneumothorax, and other AEs may occur during the application of Cabozantinib (22). Finally, TAS-115 may cause increased aspartate aminotransferase, increased alanine aminotransferase, increased blood creatine phosphokinase, increased amylase, decreased neutrophil count, decreased platelet count, decreased white blood cell count, hypophosphatemia, anemia, rash, and fever (23). The drug-related serious AEs of different TKIs in the treatment of osteosarcoma patients were basically similar. However, the AEs of PD-1 inhibitors in the treatment of osteosarcoma patients still need to be clarified.

**Table 4:** Treatment-related adverse events.

Author	Drug name	Patient No.	AEs (Grade<III)	AEs (Grade≥III)
Boye K, et al.	Pembrolizumab	7	_	Anemia, Alkaline phosphatase increase, Medullary compression, Pneumothorax, Tumorrelated pain
Le Cesne A, et al.	Pembrolizumab + cyclophosphamide	16	Nausea, Anaemia, Anorexia, Fatigue, Arthralgia, Constipation, Leucopenia, Thrombocytopenia, Weight loss, Acute renal failure, Chronic renal failure, Diarrhoea, Dyspnoea, Dysthyroidism, Lymphopenia, Neutropenia, Pruritus, Vomiting	Anaemia, Fatigue, Leucopenia, Acute renal failure, Dyspnoea, Lymphopenia
Davis LE, et al.	Regorafenib	22	Hand-foot skin reaction, Hypertension, Nausea, Diarrhea, Oral mucositis, Maculopapular rash, Vomiting, Thrombocytopenia, Hypophosphatemia, Extremity pain	Hand-foot skin reaction, Hypertension, Diarrhea, Maculopapular rash, Thrombocytopenia, Hypophosphatemia, Extremity pain
Gaspar N, et al.	Lenvatinib	31	Abdominal pain, Back pain, Neck pain, Acne, Diarrhea, Hypertension, Hypertriglyceridemia, Hyperuricemia, Insomnia, Pyrexia, Thyroglobulin increase, Pneumothorax	Pneumothorax, Arterial thrombosis, Hypertension, Diarrhea, Proteinuria, Weight loss, Abdominal pain
Xie L, et al.	Apatinib	33	Myalgia/arthralgia, Bilirubin increase, Gallbladder obstruction, Proteinuria, Hyponatremia, Hypochloridemia, Skin hypopigmentation, Hypokalemia, Hypoalbuminemia, Abdominal cramps, Diarrhea, Hypertriglyceridemia, Hypertension, Hypercholesterolemia, Hypothyroidism, Oral mucositis, Bleeding, Fatigue, Weight loss, Anorexia, Urinary frequency, Pancreatitis, Pneumothorax, Rash acneiform, Wound dehiscence, Palmar-plantar erythrodysesthesia syndrome, Pleuritic pain, Thrombocytopenia, Back pain, Anal ulcer, Palpitations, Tinnitus, Anorectal infection, Aminotransferase increase, Insomnia	Bladder perforation, Cholecystitis, Proteinuria, Hypokalemia, Abdominal cramps, Diarrhea, Hypertriglyceridemia, Anal fistula, Fatigue, Weight loss, Anorexia, Pneumothorax, Rash acneiform, Anemia, Wound dehiscence, Palmar-plantar erythrodysesthesia syndrome, Back pain, Palpitations, Pleural infection
Duffaud F, et al.	Regorafenib	29	Anaemia, Lymphopenia, Thrombocytopenia, Diarrhoea, Nausea, Constipation, Abdominal pain, Dry mouth,Vomiting, Stomatitis, Fatigue, Mucosal inflammation, Chest pain, Fever, Weight decreased, Blood alkaline phosphatase increase, Lymphocyte count decreased, Transaminases increased, Blood bilirubin increased, Gamma-glutamyltransferase phosphatase increase, Hyperbilirubinaemia, Decreased appetite, Hypophosphataemia, Muscle spasms, Back pain, Myalgia, Headache, Dysgeusia, Anxiety, Proteinuria, Dysphonia, Dyspnoea, Cough, Hand–foot skin reaction, Other skin toxicity, Hypertension	Lymphopenia, Diarrhoea, Constipation, Fatigue, Chest pain, Blood alkaline phosphatase increase, Transaminases increased, Hyperbilirubinaemia, Lipase increase, Decreased appetite, Hypophosphataemia, Hypokalaemia, Epilepsy, Dyspnoea, Pleural effusion, Haemothorax, Hand–foot skin reaction, Other skin toxicity, Hypertension
Italiano A, et al.	Cabozantinib	45	Fatigue, Diarrhoea, Oral mucositis, Hypothyroidism, AST increase, ALT increase, Nausea, Anorexia, Hair colour changes, Abdominal pain, Palmar-plantar syndrome, Thrombocytopenia, Dry skin, Weight loss, Hypophosphataemia, Neutropenia, Dysphonia, Alopecia, Hypomagnesaemia, Anaemia, Vomiting, TSH increase, Dysgeusia, Hypokalaemia, Headache, Proteinuria, Skin hypopigmentation, Constipation, Gastroesophageal reflux disease, Myalgia, ALP increase, Erythema multiforme, Lipase increased, Pneumothorax, Dry mouth, Hypocalcaemia, Epistaxis, Leucopenia, Hypertension, Dysphagia, Hyperbilirubinaemia, CPK increase, Cough, Maculopapular rash	Fatigue, Diarrhoea, Oral mucositis, AST increase, ALT increase, Palmar-plantar syndrome, Thrombocytopenia, Hypophosphataemia, Neutropenia, Hypomagnesaemia, Anaemia, Lipase increased, Pneumothorax, Leucopenia, Hypertension
Kawai A, et al.	TAS-115	20	Neutrophil count decreased, Platelet count decreased, Aspartate aminotransferase increased, White blood cell count decreased, Face oedema, Fatigue, Alanine aminotransferase increased, Hypophosphataemia, Anaemia, Rash, Pyrexia, Nausea, Diarrhoea, Blood creatine phosphokinase increased, Lipase increased, Amylase increased, Blood lactate dehydrogenase increased	Neutrophil count decreased, Platelet count decreased, Aspartate aminotransferase increased, White blood cell count decreased, Alanine aminotransferase increased, Hypophosphataemia, Anaemia, Rash, Blood creatine phosphokinase increased, Pyrexia, Amylase increased
Gaspar N, et al.	Lenvatinib with etoposide + ifosfamide	42	Nausea, Vomiting, Hypothyroidism, Diarrhoea, Pyrexia, Abdominal pain, Headache, Proteinuria, Arthralgia, Constipation, Decreased appetite, Fatigue, Asthenia, Back pain, Cough, Epistaxis, Hypertension, Oropharyngeal pain, Weight loss, Haematuria, Pain in extremity, Stomatitis, Alanine aminotransferase increased, Dizziness, Rash, Abdominal pain upper, Anaemia, Aspartate aminotransferase increased, Blood thyroid stimulating hormone increased, Alopecia, Anal fissure, Dry skin, Dysphonia, Haematochezia, Myalgia, Oral pain, Palmar-plantar erythrodysesthesia syndrome, Pneumothorax, Procedural pain, Proctalgia, Sinus bradycardia, Tachycardia, Anxiety, Bone pain, Musculoskeletal pain, Anal inflammation, Dehydration, Blood bilirubin increased, Dyspnoea, Hypophosphataemia, Lipase increased, Non-cardiac chest pain, Blood potassium decreased, Gastroenteritis, Hyperkalaemia, Hypokalaemia, Muscle spasms, Neuralgia, Pneumonia, Rectal haemorrhage, Thrombocytopenia, Toxic encephalopathy	Nausea, Vomiting, Diarrhoea, Abdominal pain, Proteinuria, Asthenia, Back pain, Cough, Epistaxis, Hypertension,Weight loss, Pain in extremity, Stomatitis, Alanine aminotransferase increased, Anaemia, Pneumothorax, Anxiety, Bone pain, Musculoskeletal pain, Anal inflammation, Blood bilirubin increased, Dehydration, Dyspnoea, Hypophosphataemia, Lipase increased, Non-cardiac chest pain, Blood potassium decreased, Gastroenteritis, Hyperkalaemia, Hypokalaemia, Muscle spasms, Neuralgia, Pneumonia, Rectal haemorrhage, Thrombocytopenia, Toxic encephalopathy, Accidental overdose, Blood magnesium decreased, Electrolyte imbalance, Eyelid oedema, Haemorrhagic diarrhoea, Febrile neutropenia, Lymphopenia, Blood count decreased, Generalised tonic-clonic seizure, Hypotension, Leukopenia, Lower respiratory tract infection, Lymphocyte count decreased, Malignant neoplasm progression, Neutropenia, Oesophageal candidiasis, Pancytopenia, Phantom pain, Renal failure, Spinal cord compression, Syncope, Tumor pain, Urticaria, Vascular device infection, Veno-occlusive disease, Ventricular dysfunction, Vulvitis, White blood cell count decreased
Xie L, et al.	Apatinib + Camrelizumab	43	Wound dehiscence, ALP increased, Blood bilirubin increased, Hypertriglyceridemia, Anorexia, Weight loss, Pneumothorax, Platelet count decreased, Diarrhea, Hand-foot syndrome, Pain in extremity, AST increased, ALT increased, Leukopenia, Rash, Mucositis oral, Hypertension, Abdominal pain, Toothache, Non-cardiac chest pain, Hypothyroidism, Blood LDH increased, Proteinuria, Cough, Nausea, Vomiting, Hair color changes, Hemorrhoidal hemorrhage, Fatigue, Peripheral neuroinflammation	Wound dehiscence, ALP increased, Blood bilirubin increased, Hypertriglyceridemia, Anorexia, Weight loss, Pneumothorax, Platelet count decreased, Diarrhea, Hand-foot syndrome, Pain in extremity, AST increased, ALT increased, Leukopenia, Rash, Mucositis oral, Hypertension, Abdominal pain, Toothache, Non-cardiac chest pain, Hypothyroidism, Blood LDH increased, Proteinuria, Cough, Nausea, Vomiting, Hemorrhoidal hemorrhage, Fatigue, Peripheral neuroinflammation

AEs (Grade<III), adverse events (drug-related, below grade 3). AEs (Grade≥III), adverse events (drug-related, grade 3 or higher).

## Discussion

Osteosarcoma is a commonly diagnosed malignant bone cancer with unclear pathogenesis. The growth factors may play important roles in the oncogenesis of this disease. Patients with refractory or relapsed osteosarcoma have a very poor prognosis. The PD-1 inhibitors and TKIs offer the few available alternatives when the surgical resection and chemoradiotherapy are not suitable.

PD-1 inhibitors are known to prevent the interaction between T-cell/PD-1 and tumor cell/PD-L1, leading to restoration of the T-cell mediated anti-tumor immunity (29). Pembrolizumab is a humanized monoclonal IgG4 kappa anti-PD-1 antibody, which has been applied in many types of malignant tumors or is undergoing clinical trials, including bladder, breast, colorectal, esophagus, gastric, head and neck, hematology, lung, melanoma, ovarian, pediatric, and other solid malignant tumors (30). Studies have shown that pembrolizumab responds better to lung metastases than other site metastases, with a 1-year survival rate of 89% for lung metastases, while the median OS of patients with recurrence and other site metastases is 6-8 months or less (31, 32). The included clinical trials reported that the median OS of advanced osteosarcoma patients treated with pembrolizumab only was 6.60 months(95% CI, 3.80-9.30) (19), while treated with pembrolizumab and low-dose chemotherapy drugs was 5.60 months (95% CI, 2.10-12.10) (20), suggesting that these patients treated with pembrolizumab did not benefit from the combination of low-dose chemotherapy drugs. Chemotherapy drugs can facilitate anti-tumor responses, such as cyclophosphamide, can increase tumor cell immunogenicity by activating CD8+ T cells and other immune cells (33). A multi-center phase II study on the treatment of advanced osteosarcomas with pembrolizumab combined with metronomic cyclophosphamide was conducted, and the results showed some benefits for patients (20). But, considering the similar demographic characteristics, drug dosage, patient’s condition and Adverse events of grade 3 or higher in these two trials, At present, the superiority of combined chemotherapy cannot be determined, and it is still necessary to refine the drug selection, dosage, and timing of application for combined chemotherapy.

Changes in tyrosine kinase receptor pathways (including VEGF, FGF and PDGF) are related to the growth, invasion and metastasis of osteosarcoma (34). VEGF expression is associated with poor long-term prognosis in osteosarcoma. FGF and FGF receptor signaling pathway promotes the resistance of osteosarcoma to radiotherapy, chemotherapy and molecular targeted therapy (35, 36). Therefore, TKIs suppress the tumor angiogenesis and induce the tumor cell apoptosis, thus to inhibit the growth and metastasis of tumor cells by inhibiting the abnormal activity of tyrosine kinase through single or multiple targets via the four signaling pathways (JAK/STAT, RAS/RAF/MEK/ERK, PI3K/AKT/mTOR, and PLC/PIP2/DAG/PLK) (14). Five TKIs including Regorafenib, Lenvatinib, Apatinib, Cabozantinib and TAS-115 were evaluated in this study.

Regorafenib is a TKI affecting the vasculature and tumor microenvironment by targeting the specific kinase proteins (VEGFR1,2,3, PDGFR, FGFR, KIT, BRAF, and RET), which is a category 1 option recommended by the NCCN guidelines for the treatment of patients with recurrent/refractory or metastatic osteosarcoma. Regorafenib showed high activity in osteosarcoma and soft tissue sarcoma and prolonged the PFS (37–39). Regorafenib was used as the test drug to treat osteosarcoma in two separated double-blind control trials by Davis (21) and Duffaud (24), which showed a similar OS. However, the study by Davis showed that the OS was 11.10 months (95% CI, 4.7-26.7) and the PFS was 3.6 months (95% CI, 2.0-7.6) in the test group; the OS was 13.4 months (95% CI, 8.5-38.1) and the PFS was 1.7 months (95% CI, 1.2-1.8) in the placebo control group; the study by Duffaud (25) showed that the OS was 11.3 months (95% CI, 5.9-23.9) and the PFS was 3.8 months (95% CI, 1.8-6.37) in the test group; the OS was 5.9 months (95% CI, 1.3-16.4) and the PFS was 1 month (95% CI, 0.7-1.33) in the placebo control group. These two studies showed that regorafenib did prolong the PFS, but the effect on prolonging OS was contradictory.

Lenvatinib is a novel antiangiogenic and orally acting multikinase inhibitor that targets VEGFR 1-3, FGFR 1-4, PDGFR-α, RET and KIT products (40). Lenvatinib treatment significantly improved the clinical outcomes (PFS and ORR) of patients with unresectable HCC compared with Sorafenib (41). In the two included articles on Lenvatinib, the children and adolescent populations were involved. From the experimental results, it can be seen that the effect of using Lenvatinib alone to treat osteosarcoma on OS and PFS is similar to the results of other trials using TKIs, which may further suggest that TKIs have little difference in treatment for different ages (as other included literature on TKIs also involves the adolescent population). But when Lenvatinib combined chemotherapy appears to be relatively beneficial for prolonging OS and PFS (**Table 1**; **Figures 2**, **3**), TKIs combined chemotherapy for osteosarcoma may be a promising option, but may also face more side effects (**Table 3**). Apatinib inhibits VEGFR-2, thereby potently reducing tumor angiogenesis and decreasing microvessel density in tumors. Apatinib showed positive results in the treatment of gastric cancer, breast cancer, lung cancer, esophageal cancer and other tumors (42). The median PFS and median OS were 5.0 months (95% CI, 3.6-6.4) and 16 months (95% CI, 14.6-17.4) in a multicenter, single-arm, prospective phase II study of Apatinib for the treatment of recurrent nasopharyngeal carcinoma (43), however, the median PFS and OS of Apatinib in osteosarcoma treatment were 4.5 months (95% CI, 3.47-6.27) and 9.87 months (95% CI, 7.97-18.93), respectively (23). Differences between the results of these two studies may be related to the age and tumor type of patients. In this study, we found that Apatinib combined with PD-1 inhibitor (Camrelizumab) seemed to have more advantages in prolonging PFS of osteosarcoma patients, this may be related to the VEGFR inhibitors improved immunotherapeutic activity of PD-1/PD-L1 antibodies by enhancing the tumor infiltration of immune cells and reducing the immunosuppressive effects of myeloid-derived suppressor cells (17), however, the risk of serious AEs is also increasing. In another trial of Apatinib combined with camrelizumab for the treatment of triple negative breast cancer (44), the PFS of continuous dosing cohort was 3.7 months (95% CI 2.0-6.4), while that of intermittent dosing cohort was 1.9 months (95% CI 1.8-3.7), which was similar to the results of the trial included in this study. However, there is currently a lack of reliable evidence to support the priority choice of TKIs combined with PD-1 inhibitors for the treatment of advanced osteosarcoma. Cabozantinib, a TKI targeting VEGFR2 and MET (a transmembrane tyrosine kinase receptor), decreased the proliferation and migration of osteosarcoma cells, and the production of RANK ligands via inhibition of the ERK and AKT signaling pathways (45). Cabozantinib improved the OS rate and prolonged the PFS of patients with advanced renal cell carcinoma or differentiated thyroid carcinoma (46, 47). Application of Cabozantinib showed that the 4-month progression-free survival was 71% (95% CI 55-83) (25). The 21 of 41 patients had tumor shrinkage and 7 patients had objective response. The tumor shrinkage rate of the targeted therapy for osteosarcoma was the highest until now. TAS-115 is an oral multikinase inhibitor targeting the MET proto-oncogene, VEGFR, and colony-stimulating factor 1 receptor, which helps to inhibit the tumor growth and the osteoclast differentiation. TAS-115 showed an anti-tumor activity (bone scan index, BSI) in a clinical trial of prostate cancer bone metastasis, which alleviated the bone pain by reducing osteoclast activity and contributed to the improvement of quality of life (48). TAS-115 also reduced the BSI and improved the progression free rates in patients with advanced osteosarcoma (26).

Both PD-1 inhibitors and TKIs show AEs, which are within the controllable range through drug withdrawal or dose adjustment. Fatigue, blood cell abnormalities and enzyme abnormalities are the most common, but none of them have drug-related lethal reactions. However, we should always pay close attention to the AEs of immune checkpoint inhibitors.

Since the most reliable efficacy information about the test drugs is provided by the randomized controlled clinical trials, limitation of this study is existed because several non-randomized clinical trials were included. Therefore, PD-1 inhibitors and TKIs in the treatment of advanced osteosarcoma still need to be further investigated by high-quality randomized controlled clinical studies.

## Conclusion

Compared with PD-1 inhibitors, TKIs showed a comparative advantage in improving the OS and are more beneficial in prolonging the PFS of patients with advanced osteosarcoma. Moreover, PD-1 inhibitors combined with TKIs is more prospective then PD-1 inhibitors or TKIs only in the treatment of advanced osteosarcoma patients, but more serious AEs may occur. We should always pay attention to the adverse drug reactions. It is still a long way to go to develop and explore new drugs with high efficiency and low toxicity.

Compared with PD-1 inhibitors, TKIs seem to have a relative advantage in improving OS and prolonging PFS, but there is currently no evidence to suggest that TKIs are superior to PD-1 inhibitors in treating advanced osteosarcoma. In addition, PD-1 inhibitors combined with TKIs or TKIs combined with chemotherapy in the treatment of advanced osteosarcoma are worth further exploration and have potential applications, but more serious AEs may occur. We should always pay attention to the adverse drug reactions. There is still a long way to go to develop and explore new drugs or combination formulations with high efficiency and low toxicity.

## Author contributions

Research: BS, XS. Article writing: BS. Interpretation of data: BS, JC, PZ. Software application: XM, CZ. Article guidance and control: YW, YY. All authors contributed to the article and approved the submitted version.
